# Fibroma and Fibromyoma of the Uterus

**Published:** 1909-03-27

**Authors:** J. Inglis Parsons

**Affiliations:** Surgeon to the Chelsea Hospital for Women


### Hospital Clinics.

### FIBROMA AND FIBROMYOMA OF THE UTERUS.

By J. INGLIS PARSONS, M.D., M.R.C.P., M.R.C.S.; Surgeon to the Chelsea Hospital for Women.

No disease of woman presents more variety than fibroma. The shape, size, consistency, number, and position may be different in each case. Further, the age of the patients may vary as much as twenty years, while in the social scale they may belong to the lowest or the highest. It must be evident from this that any attempt to lay down one line of treatment for all cases is impossible.

However much we may plume ourselves on the low mortality now obtained by hysterectomy (3¼ per cent. at the Chelsea Hospital for Women), the fact remains that to get rid of her tumours by this operation a woman has also to get rid of her organ of reproduction. To many women in the prime of life who are normal and have a natural instinct for maternity the remedy is worse than the disease. Every possible means, therefore, of treating these tumours so as to save the reproductive organs should be carefully considered. Unfortunately we know no more about the cause of fibroma than we do about cancer. Although it is not fatal to life as a rule, it is almost as great a scourge as cancer. In point of numbers it probably far exceeds that disease. Instead of being chiefly confined to the autumn of life as cancer is, fibroma most frequently attacks women in their prime, when their highest function, that of reproduction, should be at its best.

There is one fact in connection with fibroma that is of great importance, namely, its association with sterility. Either the patients have never had children and are single women, or if married they may have had one or two children and then remained sterile for some years. This naturally suggests that sterility is one of the causes of fibroma. It has been suggested that the reverse obtains, and that fibromata cause the sterility. This I do not agree with, because quite a large percentage of single women with fibroma become pregnant after marriage.

The point is one of very great importance, because during pregnancy the tumours enlarge with the uterus, and again undergo involution after parturition, and in most cases, *not all*, the tumours become smaller after the child is born, and cease to grow if the mother continues to have children. If, in the case of a young woman under thirty, the fact of leading a single life is a cause of the disease, we ought certainly to advise marriage rather than operation, particularly if after parturition we can expect a cessation of growth, and perhaps some atrophy of the tumours.

It is hardly necessary to say that such advice must be given with discrimination, that it is not applicable to patients with fibroma low down in the pelvis that might block delivery of the child. Nor is it applicable to patients with large tumours. As a rule, in *women under thirty*, the tumours are small. The typical case is a woman with one or two small tumours causing pain or menorrhagia or both.

Some eight years ago a woman, aged 25, implored me to remove her ovaries or uterus on account of the almost unbearable pains she suffered during menstruation. I found two small fibroids in the body of the uterus. A course of electricity banished the pains. She followed my advice and married. Within the year she was delivered of a fine child without any trouble, and since then has not had any symptoms from the fibroids.

It is in the early stage of fibroids that the best results are obtained by electricity for two reasons: first, the tumours conduct the current better, and, secondly, when it is concentrated on a small tumour, electricity has more effect on its vitality than when diffused over a large one. In suitable cases we may expect to stop the menorrhagia and the pains and the growth for four to five years in 80 per cent. of the cases treated. After that time the treatment may have to be repeated.

Another patient, aged 23, was sent for profuse menorrhagia due to a fibroid polypus. This was removed by operation *per vaginam*, and a small fibroid was also discovered in the body of the uterus. Removal of the polypus stopped the menorrhagia. She married, and soon had a child without difficulty, except that a piece of placenta adhered to the uterine wall over the site of the fibroid and had to be curetted.

The risk which patients run during parturition from the presence of fibroids has been much exaggerated. Provided the tumours are not large, and do not interfere with the passage of the child through the pelvis, the majority of cases do quite well.

In another instance the tumour was subperitoneal. The patient was aged 24, and was sent to me by Dr. Keightly for dysmenorrhœa. A tumour could be felt to the right of the uterus and appeared to be a small ovarian fibroma. On opening the abdomen it was found to be a small fibroma projecting from the right corner of the uterus. After myomectomy the patient recovered and her symptoms disappeared.

In addition to the use of electricity and myomectomy in young women with small tumours, a good deal can be done by medical treatment, so as to avoid removing the uterus and to allow time for pregnancy to occur. The most potent drug is thyroid extract. It is, of course, necessary to see the patient at regular intervals during the treatment so as to reduce the dose or withhold it if symptoms of thyroidism supervene. As a rule small doses do best continued for two or three months. The results obtained vary as one might expect, since the tumours also vary in consistence, size, structure, and position.

Saline aperients relieve the uterine congestion and always do good. On the other hand it is quite a mistake to give iron except as a temporary measure. When the menorrhagia is producing anæmia attempts must be made to stop the menorrhagia. A patient taking iron makes more blood and then loses it at the next menstruation. The result is that after a definite time the recuperative power of forming new corpuscles is to a great extent exhausted, and the patient relapses into a condition of permanent anæmia. Should hysterectomy become necessary it is most important that we should be able to tone up the patient so as to stand the operation. This is very difficult if her blood-forming powers are to a great extent exhausted. When, therefore, the menorrhagia persists in spite of all remedial measures the situation must be faced and operation advised before the patient's strength has all drained away.

Of the other drugs ergot is of no value in my experience, nor is styptol. The endometrium in fibroma is much thickened by proliferation of the normal glands, so that in many cases it forms what may be called a layer of adenomatous growth, much more vascular than the normal endometrium. It is this which causes the metrorrhagia.

Chloride of calcium is of undoubted value by increasing the coagulability of the blood. It is apt to cause constipation, which is rather a drawback. One patient to whom I gave this drug noticed herself a diminution of something like 30 per cent. in the loss at menstruation whenever she took it.

When the tumours have attained a good size and the patient is over 30, the problem assumes a different aspect. In the first place, a woman's most fertile years are between 20 and 30. If she has been married some years and has not conceived, she is never likely to. Therefore there is not the same reason for preserving her uterus as there would be in a younger woman with a small tumour who would probably bear children.

As the patient's age approaches 40 or beyond, another factor cames into play, namely, the onset of the menopause. There is no doubt that the average age for the climacteric is more than 45, and when fibroid tumours are present a large majority continue to menstruate until they are 50 and even older. Taking then a woman of 40 we cannot expect her to derive any benefit for probably ten years by the onset of the menopause.

Another point is that if we perform hysterectomy and remove the whole of the body of the uterus the menopause will follow although both ovaries and tubes are left behind. There is no great objection to this in a woman about 40. She bears it quite well and with but little more disturbance than if the menopause had come on naturally. But in a woman under 30 the forced onset of the menopause often causes grave disturbance of the nervous system with a tendency to melancholia.

Palliative treatment for large tumours is not so beneficial. In regard to electricity the results are not so good. A current which will easily affect the nutrition of a tumour having a cubic capacity of say four inches will have very much less effect when spread over forty cubic inches. Large tumours are generally of greater age than small, and for this reason often contain more fibrous tissue. Now dense fibrous tissue offers a greater resistance to the passage of electricity than the normal tissues. Consequently the current may not pass through the tumour at all. It may, therefore, remain quite unaffected by the treatment. In order to stop the menorrhagia it is necessary to apply the electrode to the uterine cavity. There is very often so much distortion that this cannot be done.

For these reasons the best treatment for women over 30 with large tumours is hysterectomy. The nearer they are to 40 the stronger the reasons become.

The risk of the operation is now very small; hardly, if at all, more than that from ovariotomy, provided rigid asepsis is carried out and also that the patient's resisting power is raised by appropriate treatment beforehand. Perhaps the most important point is to tone up the patient's heart, which is often enfeebled, not only by the anæmia but also apparently by the growth of the tumours: for this purpose the best drug is strychnine. A week or ten days' rest in bed before the operation is also of great benefit.

In doing this operation it is quite unnecessary to have half a dozen pairs of forceps flopping about and bruising the tissues in the pelvis. The outer half of the broad ligament is tied with silk, and the inner on the tumour held by a pair of Doyen's forceps. Both ligaments are then split down as far as required. The bladder is carefully separated by cutting across the peritoneum. The mass is then cut across about the level of the internal os while the assistant clamps the uterine arteries and continues to hold them until the vessels are tied. The stump of the cervix is then drawn together with mattress sutures so as to prevent any subsequent oozing. A continuous suture unites the cut ends of the peritoneum and covers over the whole of the raw surface.

The difficult cases are those where the tumours grow low down in the cervix and project into the broad ligament. Under these conditions the ureter is very likely to be lifted up and lie on the tumour or adhere to it, or the bladder is drawn right up on to the tumour, or it may be entirely separated from the uterus and adherent to the fibroma. This happened to one of my cases, a huge fibroma of the broad ligament. In removing the tumour the bladder was opened. It was sewn up and the patient made a good recovery. Sometimes a tumour is impacted in the pelvis and held down by adhesions. If these are very dense it is best to leave them to the last, tie off the broad ligaments and the uterine arteries if possible, and then dissect off a thin shell from the capsule of the tumour. By this method the adhesions are not disturbed and there is no risk of opening the bowels. It may be very difficult to cut across the uterus if it is fixed low down and cannot be drawn up. Sometimes a tumour fills the brim of the pelvis on each side, while the broad ligaments are low down and can neither be seen or felt. By forcibly rotating the uterus from side to side we can usually apply forceps and divide them, when the tumours can be further drawn up and the operation proceeded with.

It is not uncommon to find signs of degeneration in one or more of the tumours removed by hysterectomy, and the presence of this is by some operators considered to justify the operation. As a matter of fact degenerating tumours have ceased to grow, and are undergoing spontaneous cure. It is not these but the sound growing tumours that justify the operation. I have in many patients watched the shrinking of tumours undergoing degeneration at the menopause and after without any symptoms or inconvenience being caused by it. That degeneration is not dangerous is proved by the fact that when in past years there was no relief by operation it was quite uncommon for death to occur.

Fibroids rarely slough unless pedunculated and projecting into the uterine cavity. A pedunculated tumour within the abdomen rarely sloughs. It is much more likely to form adhesions and take on new vessels, which help to keep it alive and may provide its chief blood supply. If, however, sloughing does occur, operation is necessary to prevent septicæmia.

Should the cervix be left *in situ* or removed? It has been said that carcinoma is liable to attack the stump, but no one has brought forward a series of cases to demonstrate it. Carcinoma may occur with fibromata of the uterus, but it is distinctly uncommon; further, for carcinoma to begin in the fibroma itself is extremely rare.

On the other hand, the operation can be done more quickly and with greater safety if the cervix is not removed. It also makes a better roof to the vagina, and is, on the whole, more comfortable.

Some patients even with large tumours and suffering great pain and discomfort absolutely refuse operation. What can be done then? I have already pointed out that the percentage of successes from electrical treatment is nothing like so high with large tumours as with small. At the same time it is worth trying if operation is refused. On looking over my notes of cases I find that something like 60 per cent. become very much better. For instance, in 1896 Dr. Hames brought Mrs. E., aged 46, to see me. She was suffering from severe menorrhagia and continual pains in the hypogastrium, aggravated by defæcation. She had two children, the youngest aged twenty years. The tumour had been diagnosed in 1892 and operation refused. On examination I found a large fibromatous mass extending up to the umbilicus, involving the cervix, and measuring six to seven inches across above the brim of the pelvis. The sound passed easily forwards 4½ inches. The uterine cavity lay in the front of the mass.

Twenty-two applications of electricity were given with an average strength of 120 milliamperes for 15 minutes twice a week. The profuse loss was reduced to one-half and the pains disappeared.

Four years later—in 1900—she became irregular, and at the end of another year menstruation had stopped entirely. Since then the mass has shrunk in size. I have seen her quite recently and she has no trouble whatever from the tumours.

Another patient who refused operation was sent to me by Dr. Vernon Jones in 1896. Mrs. B., aged 41, complained of severe menorrhagia, pain, and distension of the abdomen. The uterus, enlarged by fibromata, extended to within three inches of the umbilicus. Thirty-three applications of electricity were given. The loss was reduced to normal, the pains disappeared, and the tumour became smaller. In 1904 the menopause began. The tumours are now half the size they were in 1896, and the patient feels very well.

Sometimes electricity fails to relieve the pains and arrest the growth, probably because the tumours in certain cases offer a greater resistance to the passage of the current than the normal tissues. Even then it generally improves the patient's health, and enables her to stand the operation better.

In 1904 Dr. Keightley sent me a patient, aged 40, with a large fibroma impacted in the pelvis, continued metrorrhagia, constant pain, and wasting. After twenty-four applications she had put on a stone in weight. The pains, metrorrhagia, and indigestion had disappeared. She remained well for three years. In 1907 the treatment was repeated as there were indications of a return of pain. She is now very well, and the fibroid has decreased in size by one-third. She is very pleased with herself for having absolutely refused operation.

Thyroid extract sometimes does good in large tumours also as the following notes show. A nurse, aged 42, was obliged to give up nursing on account of constant pain in the pelvis due to multiple fibromata forming a mass that extended some inches above the brim of the pelvis. On account of the distortion of the uterine canal it was impossible to pass an electrode. Menstruation was fairly regular and not excessive, but was very painful. After taking thyroid extract for six months her pains had gone, the tumour was smaller, and she was able to resume work. She also had refused operation. Considering the general low condition of her health I doubt if she would have been able to work any sooner even if hysterectomy had been done. At the present time she is going on quite well, and the menopause is beginning, while the fibroids are smaller than before.

One great drawback to the electrical treatment is that it takes up so much of the surgeon's time without adequate remuneration compared to operation, and it is nothing like so easy to carry out successfully as a hysterectomy. This no doubt accounts in a measure for the greater popularity of operation among surgeons, and my sympathies are with them.

